# Metacognition-augmented cognitive remediation training reduces jumping to conclusions and overconfidence but not neurocognitive deficits in psychosis

**DOI:** 10.3389/fpsyg.2015.01048

**Published:** 2015-08-03

**Authors:** Steffen Moritz, Teresa Thoering, Simone Kühn, Bastian Willenborg, Stefan Westermann, Matthias Nagel

**Affiliations:** ^1^Clinical Neuropsychology, Department of Psychiatry and Psychotherapy, University Medical Center Hamburg-EppendorfHamburg, Germany; ^2^Center for Lifespan Psychology, Max Planck Institute for Human DevelopmentBerlin, Germany; ^3^Department of Psychiatry and Psychotherapy, University of LübeckLübeck, Germany; ^4^Department of Clinical Psychology and Psychotherapy, Institute of Psychology, University of BernBern, Switzerland; ^5^Asklepios Medical Center Hamburg-North–Wandsbek, Department of Psychiatry and PsychotherapyHamburg, Germany

**Keywords:** psychosis, schizophrenia, neurocognition, cognitive biases, cognitive remediation

## Abstract

The majority of patients with schizophrenia display neurocognitive deficits (e.g., memory impairment) as well as inflated cognitive biases (e.g., jumping to conclusions). Both cognitive domains are implicated in the pathogenesis of the disorder and are known to compromise functional outcome. At present, there is a dearth of effective treatment options. A total of 90 patients with schizophrenia were recruited online (a diagnosis of schizophrenia had been confirmed in a large subgroup during a previous hospital admission). Subsequent to a baseline assessment encompassing psychopathology, self-reported cognition as well as objective memory and reasoning tests, patients were randomized to one of three conditions: standard cognitive remediation (mybraintraining), metacognition-augmented cognition remediation (CR) condition (variant of mybraintraining which encouraged patients to reduce speed of decision-making and attenuate response confidence when participants made high-confidence judgements and hasty incorrect decisions) and a waitlist control group. Patients were retested after 6 weeks and again 3 months after the second assessment. Groups did not differ on psychopathology and neurocognitive parameters at any timepoint. However, at follow-up the metacognitive-augmented CR group displayed a significant reduction on jumping to conclusions and overconfidence. Treatment adherence correlated with a reduction of depression; gains in the training exercises from the standard mybraintraining condition were correlated with improved objective memory performance. The study suggests that metacognition-augmented CR may ameliorate cognitive biases but not neurocognition. The study ties in well with prior research showing that neurocognitive dysfunctions are rather resistant to change; the failure to detect significant improvement of CR or metacognition-augmented CR on psychopathology and neurocognition over time may partly be attributed to a number of methodological limitations of our study (low psychopathology and chronicity of participants, low “dosage,” narrow range of tests, self-report psychopathology scales).

## Introduction

Schizophrenia is frequently accompanied by neuropsychological deficits which are spread across a wide range of cognitive functions (Heinrichs and Zakzanis, 1998; Keefe and Harvey, 2012). Memory and attention problems in concert with social cognitive impairments (Fett et al., 2011) are a major predictor for disability and low functional outcome in the disorder (Green, 1996; Green et al., 2004; Lepage et al., 2014). Neurocognitive deficits are also a risk factor for poor symptomatic outcome. First, memory problems aggravate medication non-adherence as patients may fail to remember the rationale for drug administration or forget to take their medication (Moritz et al., 2013b), particularly due to prospective memory failure (Moritz et al., 2004). In addition, compromised attention, reasoning, and memory capacity may limit the comprehension and internalization of knowledge and skills acquired during cognitive therapy and thus impede transfer to everyday life.

The causes underlying neurocognitive deficits in schizophrenia are multi-facetted. Apart from early (neurodevelopmental) deficits that already manifest prior to the onset of the disorder (Bang et al., 2014; Corigliano et al., 2014), avolition/lack of effort and a restricted non-challenging environment/hospitalization may compromise cognition. Some recent studies suggest that (conventional) antipsychotics impair brain functioning (Ukai et al., 2004; Ho et al., 2011; Gasso et al., 2012), which in turn hampers neurocognition. While antipsychotic-induced cognitive deficits are clearly non-desired and thus usually considered a side-effect, there is emerging, albeit not yet conclusive, evidence that such secondary cognitive deficits may in fact be one mechanism through which antipsychotics reduce positive symptoms (“effect by defect” hypothesis; Moritz et al., 2013a). In other words, there may be two sides of the same coin: doubt and reduced speed of information processing induced by antipsychotics may be a prerequisite for the dissolution of delusions.

Currently, there is a dearth of potent treatment options against cognitive deficits. Early claims that atypical neuroleptics may act as cognitive enhancers have not lived up to its expectations (Keefe et al., 2007; Davidson et al., 2009; Keefe and Harvey, 2012). Atypical neuroleptics leave cognition uncompromised at best. It should also be taken into account that side effects such as extrapyramidal symptoms (Fervaha et al., 2015) and concomitant medication, particularly anticholinergic drugs (Vinogradov et al., 2009) and tranquilizers/benzodiazepines (Deckersbach et al., 2011) are known to aggravate neurocognitive deficits, too.

Cognitive remediation (CR) has shown some promise; meta-analyses indicate that CR exerts a (small-to-moderate) effect on neurocognition (McGurk et al., 2007; Wykes et al., 2011) but does not have a lasting impact on symptomatology (Wykes et al., 2011). However, this promising evidence has to be weighed against the effort that needs to be invested to produce those changes (e.g., one-on-one training, tailored material). Recently, low-threshold group CR trainings have shown some beneficial effect. A meta-analysis on 36 studies reveals that Integrated Psychological Therapy (IPT), a program at the interface of neurocognition and social cognition, exerts significant positive effects relative to control interventions on neurocognition, social cognition, psychosocial functioning, and negative symptoms (Roder et al., 2011). In a recent study we were able to show that a CR group improved attention after 3 years relative to a metacognition group (Moritz et al., 2014c).

Apart from “cold” cognitive deficits mirroring brain dysfunction in psychosis, particularly in the frontal and temporal lobes, there is an emerging interest in cognitive biases. Cognitive biases are not deficits *per se* but represent alterations or styles in the perception and processing of information, for example a preference to remember positive versus negative information. Cognitive biases are not pathological as such; some cognitive biases can even promote psychological well-being (e.g., self-serving bias, “Pollyanna effect”; Bentall, 1992; Pohl, 2004). Among other cognitive distortions, studies have implicated jumping to conclusions (Garety et al., 1991) and overconfidence in errors (Moritz et al., 2003) in the formation and maintenance of psychosis. To summarize, a plethora of studies suggest that patients with schizophrenia are hastier in gathering information (for reviews, see Garety and Freeman, 1999, 2013; Fine et al., 2007) and are more confident in erroneous responses pertaining to memory (Moritz and Woodward, 2006a; Gaweda et al., 2012; Peters et al., 2013) and social cognition (Kother et al., 2012; Moritz et al., 2012b) relative to non-clinical and psychiatric controls. Recent evidence suggests that this extends to perception (Moritz et al., 2014b). Both biases foster the formation of momentous false decisions that under some contextual factors may promote delusions (Moritz and Woodward, 2006b; Garety and Freeman, 2013). To illustrate, jumping to conclusions may lead a person with a history of psychosis to infer that a friend who is not calling back within 2 days has turned his back on him and is not trustworthy anymore. This along with overconfidence in errors may later turn the initial benign suspicion into a serious false belief (e.g., that the friend is a police spy who has gathered sufficient information against the patient so that they can terminate surveillance). Once such ideas have systematized, judgments are usually not validated or questioned anymore and the person is no longer open to disconfirmatory evidence, the latter representing another prominent cognitive bias (Woodward et al., 2006, 2008; Veckenstedt et al., 2011).

The present study examined the efficacy of CR training versus a CR training combined with a bias modification approach. To this end, a low-threshold online CR training called mybraintraining Professional (from here on “mybraintraining”) was administered. Mybraintraining intends to improve neurocognitive functioning by training four major faculties: calculation, logic, memory, and vision. We set up two experimental CR conditions which were tested against a waitlist control group. In the standard CR condition, patients were encouraged to avoid making errors when performing cognitive tasks that were presented under time restriction. In the metacognition-augmented CR condition the same exercises were presented but patients additionally had to rate their responses in terms of confidence, that is, whether they were certain or not that their responses were correct. Whenever a subject made a high-confident error and/or an error committed with very short reaction time (i.e., less than half of the allocated time used) they were advised to attenuate their confidence and to take more time if not fully confident for the remaining trials. The aim of this metacognition-augmented CR condition was to sensitize participants to the disadvantages of high-confident and hasty decision-making suggesting that “gut feelings” may be faulty. We hypothesized that the conventional CR condition may improve subjective and perhaps even objective cognitive impairment. The metacognition-enhanced CR condition was hypothesized to additionally improve the jumping to conclusions bias and to attenuate response confidence (as measured by a memory task).

## Materials and Methods

### Participants

The present study was approved by the ethics committee of the German Society for Psychology (DGPs). Participants were recruited from various sources. A total of 223 former patients of the Department of Psychiatry and Psychotherapy, University Medical Center Hamburg-Eppendorf (Germany) with verified diagnostic status (schizophrenia or schizoaffective disorder) were informed about the study via email. All participants had given explicit permission to be contacted for future studies. Furthermore, 309 emails were sent to clinicians asking them to pass on information about the study to patients meeting inclusion criteria. Finally, upon the approval of webmasters study invitations were posted in several guided self-help internet networks pertaining to schizophrenia and psychosis (these websites provided reliable information on the disorder and fostered the exchange of individuals affected with psychosis).

The following inclusion criteria were applied: age between 18 and 65 years, willingness to provide electronic informed consent and to participate in anonymous (internet-based) surveys as well as a diagnosis of schizophrenia or schizoaffective psychosis.

All posts and emails contained a weblink directing interested parties to the baseline survey. The trial was created using Questback^^®^^ which does not store IP addresses. Group allocation was carried out at random.

The first page of the online survey essentially repeated the information of the email (random assignment to either the mybraintraining standard version, metacognition-augmented mybraintraining, or waitlist control group; inclusion criteria) in everyday language. It was announced that all participants would receive free access to the online program (mybraintraining) for 1 year, either immediately or after a 6-week delay. Moreover, all completers would receive a manual containing mindfulness exercises at the end of the study.

Multiple log-ins via the same computer were prevented by means of “cookies.” The survey consisted of the following parts: invitation, informed consent (mandatory), optional consent to contact the patient’s clinician in order to verify diagnostic status (to do this, participants had to provide their own name as well as the name and address of the clinician), demographic section (e.g., gender, age), medical information (e.g., medication, psychiatric diagnoses), assessment of psychopathology I (see questionnaire section below), encoding memory phase, assessment of psychopathology II (see questionnaire section below), memory recognition test, fish task (jumping to conclusions), and request for an email address (to match baseline and post survey data). Then, we asked participants to endorse whether or not they had responded honestly. Finally, participants were given the opportunity to leave comments.

No monetary compensation was offered for participation. Individuals who were randomly assigned to the waitlist condition were informed that they would receive access after completing the follow-up survey 6 weeks later.

Participants in two experimental groups were given access to one of two versions of mybraintraining within 24 h. This email also contained information about the rationale of mybraintraining or metacognition-augmented mybraintraining. Participants in the experimental groups received weekly email reminders encouraging them to use the program on a regular basis.

### Procedure

Six weeks after the baseline assessments, participants were invited via email to participate in the post survey. Up to two reminders were dispatched in case subjects failed to complete the post assessment. Three months after the post assessment, invitations for a follow-up assessment were sent. Again, up to two reminders were dispatched if subjects did not fill out the final assessment.

### Post Assessment

For the post survey, individuals were requested to enter their email address to allow matching post data with baseline data. The post assessment consisted of the following parts: introduction, current treatment and medication, assessment of psychopathology I, encoding memory phase, assessment of psychopathology II, memory recognition test, fish test (jumping to conclusions), and evaluation of the online training (see below). Similar to the baseline assessment, we asked participants whether or not they had responded honestly and gave them the opportunity to leave comments.

Subsequent to completion of the post assessment, all participants received a manual on relaxation and mindfulness exercises. Participants in the waitlist condition also received access to the standard CR condition. Patients in the standard mybraintraining condition did not receive the metacognition-augmented CR training and vice versa.

### Follow-Up Assessment

Three months after the post assessment, participants were invited to a follow-up assessment. This final assessment was not part of our initial study design. As participants in the waitlist group received access to the mybraintraining standard version subsequent to completion of the post assessment, this final follow-up assessment did not allow comparison of the three groups. Hence, the follow-up analysis compared the standard CR group (immediate or delayed) with the metacognition-augmented CR group. As an incentive for continued participation, individuals received a manual with exercises derived from “Acceptance and Commitment Therapy.” The follow-up assessment was a shorter version of the post assessment and involved a selection of previously used scales (see below). As the follow-up was not announced from the start, we expected a higher non-completion rate.

### Questionnaires (Online Assessment)

Participants were asked to complete the following questionnaires (the survey proceeded only after all items had been answered):

### Paranoia Checklist (Freeman et al., 2005)

The Paranoia Checklist is an 18 item questionnaire assessing paranoid beliefs and suspiciousness. The psychometric properties are good (Freeman et al., 2005; Lincoln et al., 2010a,b). In our slightly adapted version, participants are asked to rate their present symptom severity on a five-point Likert scale ranging from 1 (not at all) to 5 (extremely).

### Center for Epidemiologic Studies-Depression Scale (CES-D)

The Center for Epidemiologic Studies-Depression Scale (CES-D) is a 20 item questionnaire covering depressive symptoms; the reliability and validity of the CES-D have been established previously (Radloff, 1977; Hautzinger and Brähler, 1993). In the present study, CES-D items were presented intermixed with items from the Paranoia Checklist.

### Launay-Slade Hallucination Scale-Revised (LSHS-R; Bentall and Slade, 1985)

The Launay-Slade Hallucination Scale-Revised (LSHS-R) is a 16 item questionnaire covering sleep-related hallucinations, vivid daydreams, intrusive thoughts, and auditory hallucinations. Its reliability has been demonstrated elsewhere (Goodarzi, 2009). Psychosis patients with hallucinations usually score higher than remitted patients, and the latter in turn reach higher scores than patients who never experienced hallucinations (Varese et al., 2012). The LSHS-R was not included in the follow-up assessment.

### Beck Cognitive Insight Scale (BCIS) – Extended

The Beck Cognitive Insight Scale (BCIS; Beck et al., 2004) is a 15-item scale that measures the degree of patients’ self-reflectiveness and overconfidence in the interpretation of experiences. Principal component analysis (Beck et al., 2004) suggests a two-dimensional structure (self-reflectiveness and self-certainty). According to the original article (Beck et al., 2004), the BCIS demonstrates good convergent, discriminant, and construct validity. The psychometric properties of the German translation used in the present study are good as well (Mass et al., 2012). We complemented the BCIS with a number of self-developed novel items asking for subjective cognitive deficits (e.g., “I have trouble learning new things”). The BCIS was not administered in the follow-up assessment.

### Jumping to Conclusions

We administered an online version of the probabilistic reasoning task (Speechley et al., 2010; Moritz et al., 2012a), which slightly differs from the original beads task as it employs a different scenario (lakes with fish instead of jars with beads). Three parallel versions were set up to avoid practice effects. In each version, two lakes with colored fish in opposing likelihood (e.g., 80% orange vs. 20% gray fish, and vice versa) were presented to the participant. Following each “catch,” participants were asked to make two judgments: (1) a probability judgment about the likelihood that the fish was/were being caught from lake A versus lake B, and (2) whether the available amount of information would justify a decision or not. The instruction emphasized that the fisherman would not change the lake throughout the task. The ratio of fish in each lake was shown at the bottom of each slide along with previously caught fish (the last catch was indicated with an arrow). In total, 10 fish were presented; one lake was strongly suggested by the chain of events (D–D–D–N–D–D–D–D–N–D; D = dominant color of fish; N = non-dominant color of fish). Jumping to conclusions was defined as a decision after one or two fish. We also computed the number of draws to decisions.

### Memory Test

Three parallel versions of a newly developed memory recognition test were composed. The test was modeled after the Auditory Verbal Learning Memory Test (AVLT) but did not encompass active recall. In the (incidental) encoding phase (i.e., unlike in the AVLT participants were not instructed that their later task would be to memorize the items), participants were presented 15 nouns [each five words that were pre-classified by the authors as positive (e.g., cake), negative (e.g., accident) or neutral (e.g., table)] and requested to appraise each noun as either positive, neutral or negative (valence). Later, participants were presented the previously presented 15 words intermingled with 15 distractor words of different valence in random order (recognition phase). Participants were asked to rate if the respective word had been presented before (i.e., in the valence task) and how confident they were in the correctness of their judgment. Items had to be endorsed on a four-point Likert scale (1 = old word, certain; 2 = old word, uncertain; 3 = new word, uncertain; 4 = new word, certain). There was an equal number (*n* = 15) of (pre-defined) negative, positive, and neutral words, both with respect to old (studied) and new (distractor) words.

### Mybraintraining Professional

Mybraintraining is a CR program which is available online (no local installation on PC necessary) at http://www.mybraintraining.com/. The program can be used both as a self-help or conventional treatment device (i.e., guided treatment by neuropsychologist or occupational therapist). The program encompasses 30 exercises aimed at stimulating executive functioning. Exercises fall into four broad categories: calculation, logic, memory, and vision. The exercises were designed during development of the “Train your Brain with Dr. Kawashima” program in cooperation with the Industry University Research Project with Professor Dr. Ryūta Kawashima. According to the developers (personal communication), performance of each exercise had to be accompanied by activation of the frontal lobe (presented in the “Scientific Details” part of each exercise).

The difficulty of the sessions automatically adapts to the patients’ performance. mybraintraining includes motivating elements as used commonly in video games in order to increase fun and adherence. The administrator can define individual training plans and adapt exercises to each patient’s needs (e.g., level of difficulty, varied time limits, etc.). This tool also compiles statistics (e.g., to compare one patient with reference group, number of sessions completed, training success). Data protection and security comply with industry standards.

For the present study, we used the “daily test” tool of mybraintraining Professional which encourages patients to perform a random string of four exercises, one from each category (calculation, logic, memory, and vision).

**FIGURE 1:**
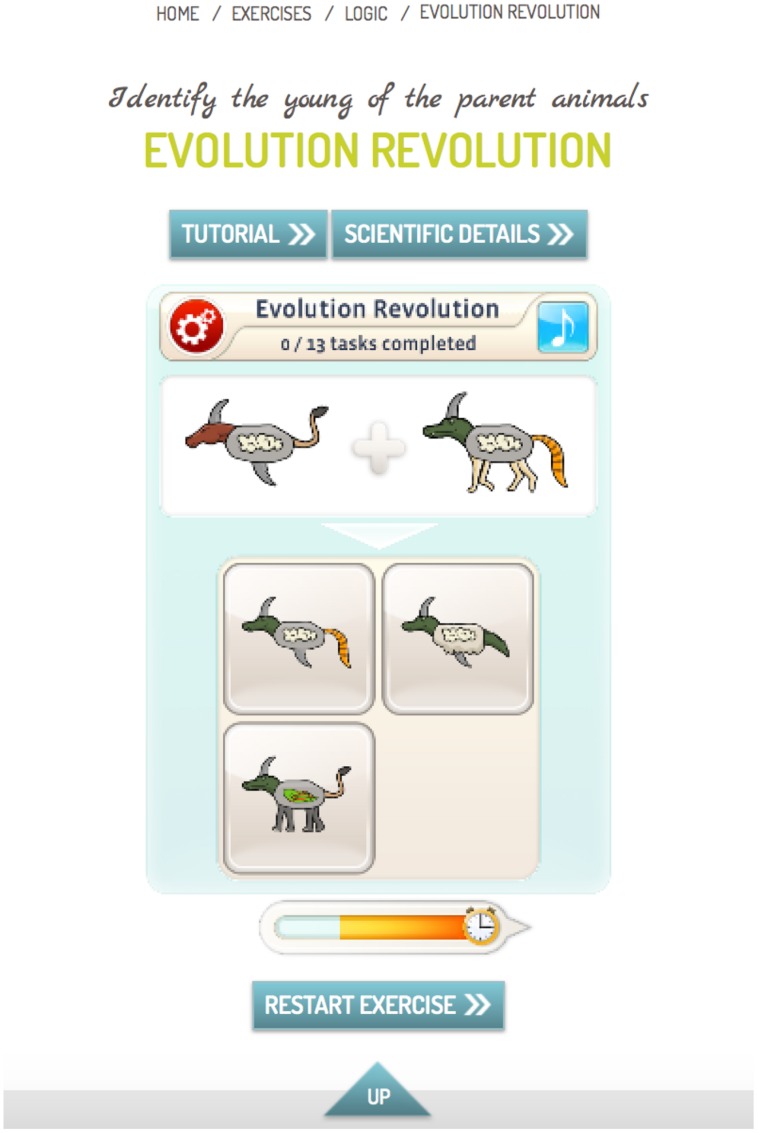
**Example for an exercise from category “Logic.”** The participant had to identify the young of the parent animals (the upper left response option is correct). In the standard version, the participant had to indicate his or her choice and was then informed about the outcome (correct versus incorrect). In the metacognitive-augmented condition, the participant was asked after each response whether he or she was certain that the response was correct. In case of a very fast incorrect response (less than half of the allotted time indicated by the time bar; see bar left to clock symbol) or a high-confident incorrect response, patients were encouraged by automatic feedback to either take more time before completing an item and/or to attenuate response confidence if the available evidence was insufficient.

In addition to the conventional version of mybraintraining Professional, a condition termed metacognitive-augmented CR condition was constructed, which aimed to reduce overconfidence and jumping to conclusions. This version asked participants to make a confidence judgment (certain versus uncertain) after each trial. The program then provided feedback in case of hasty and/or high-confident errors (see **Figure 1**). Since the termination of the study, this additional option is now part of the standard program.

### Strategy of Data Analysis

Simple cross-sectional analyses were performed using *t*-tests for metric (e.g., age) and cross table statistics for nominal data (e.g., gender distribution). For group comparisons over time we used mixed ANOVAs with Group as the between-subject factor and Time as the within-subject factor when using metric data. In case of binary data (e.g., rate of jumping to conclusions) a generalized estimating equations procedure was performed which was deemed more appropriate than a conventional repeated-measures ANOVA.

## Results

**Table 1** shows the baseline characteristics of the sample, of which 76 patients could be reached for the post assessment and 38 for the follow-up. No significant differences emerged for any demographic, psychopathological, or cognitive variable.

**Table 1:** Baseline characteristics of the full sample. Means, SD, (in brackets) and frequency.

Variable	Waitlist	Standard cognitive remediation	Metacognition-augmented cognitive remediation	Statistics
	(*n* = 30)	(*n* = 30)	(*n* = 30)	
**Background variables**				
Age in years	37.03 (12.66)	40.10 (9.29)	40.80 (9.97)	*F*(2;89) = 1.04, *p* = 0.356
Sex (male/female)	10/20	11/19	12/18	χ^2^ = 0.29, *p* = 0.866
**Parallel treatments**				
Antipsychotics (yes/no)	27/3	25/5	26/4	χ^2^ = 0.58, *p* = 0.749
Inpatient treatment (yes/no)	1/29	2/28	1/29	χ^2^ = 0.52, *p* = 0.770
Outpatient treatment (yes/no)	20/10	17/13	17/13	χ^2^ = 0.83, *p* = 0.659
Psychotherapy (yes/no)	8/22	7/23	7/23	χ^2^ = 0.12, *p* = 0.942
Waiting for psychotherapy (yes/no)	1/29	2/28	1/29	χ^2^ = 0.52, *p* = 0.770
**Reasoning**				
Jumping to conclusions (decision after first or second fish)	40%	47%	40%	χ^2^ = 0.36, *p* = 0.833
**Memory test**				
Hits	13.23 (2.31)	13.50 (2.05)	13.52 (1.23)	*F*(2;89) = 0.74, *p* = 0.483
False alarms	1.00 (1.76)	0.93 (1.46)	0.77 (1.14)	*F*(2;89) = 0.20, *p* = 0.820
High-confident responses	25.97 (5.69)	25.00 (5.41)	26.83 (3.56)	*F*(2;89) = 1.02, *p* = 0.365
**Beck Cognitive Insight Scale (extended)**				
Self-reflectiveness	9.13 (3.16)	8.90 (2.88)	10.10 (3.08)	*F*(2;89) = 1.31, *p* = 0.274
Self-certainty	7.53 (2.61)	6.77 (2.53)	6.70 (2.31)	*F*(2;89) = 1.04, *p* = 0.357
Subjective cognitive dysfunction	9.20 (3.80)	8.40 (4.06)	9.00 (4.19)	*F*(2;89) = 0.32, *p* = 0.726

Across time, medication status did not change between groups (*p* > 0.3). At baseline, 87% of the participants were medicated with antipsychotics, at post (85%) and follow-up (87%) the rate was almost identical. Likewise, treatment status [yes (i.e., outpatient, inpatient, day clinic, practitioner) versus no] did not change between groups across time (*p* > 0.5). Most patients were treated as outpatients (pre: 60%, post: 57.5%, follow-up: 53.3%). Rates did not differ among groups at any point in time (*p* > 0.6).

### Pre versus Post

**Table 2** shows between-group differences from pre to post for the per protocol sample (i.e., participants in the CR conditions had logged into mybraintraining at least once). Groups did not differ significantly on any symptoms, and cognition measures.

**Table 2:** Differences among conditions across time (sample with available pre–post scores).

Variable	Waitlist (*n* = 29)		Standard cognitive remediation (*n* = 20)		Metacognition-augmented cognitive remediation (*n* = 20)		ANOVA (G = group effect, T = time, I = interaction) [for JTC generalized linear equations were applied]


	pre	post	pre	post	pre	post	
Draws to decision	3.72 (2.34)	4.55 3.27)	3.32 (2.36)	2.84 (1.77)	3.20 (2.14)	3.70 (2.27)	G: *F*(2;65) = 1.31, *p* = 0.277, $\eta_{p}^{2}$ = 0.04 T: *F*(1;65) = 1.64, *p* = 0.204, $\eta_{p}^{2}$ = 0.02 I: *F*(2;65) = 3.09, *p* = 0.052, $\eta_{p}^{2}$ = 0.09
JTC (decision after 1st or 2nd fish = 1)	0.38 (0.49)	0.31 (0.47)	0.47 (0.51)	0.53 (0.51)	0.40 (0.50)	0.40 (0.50)	G: Wald χ^2^(1) = 0.48, *p* = 0.827 T: Wald χ^2^(1) = 0.00, *p* = 0.980 I: Wald χ^2^(1) = 0.78, *p* = 0.378
**Beck Cognitive Insight Scale – extended**							
Self-reflectiveness	8.97 (3.08)	8.72 (2.85)	8.85 (2.30)	9.95 (2.76)	9.70 (3.20)	9.10 (3.91)	G: *F*(2;66) = 0.37, *p* = 0.694, $\eta_{p}^{2}$ = 0.01 T: *F*(1;66) = 0.06, *p* = 0.813, $\eta_{p}^{2}$ = 0.00 I: *F*(2;66) = 1.87, *p* = 0.162, $\eta_{p}^{2}$ = 0.05
Self-certainty	7.72 (2.43)	6.34 (1.91)	7.05 (2.28)	6.85 (2.66)	6.90 (2.31)	6.75 (2.17)	G: *F*(2;66) = 0.06, *p* = 0.943, $\eta_{p}^{2}$ = 0.00 T: *F*(1;66) = 6.84, *p* = 0.013, $\eta_{p}^{2}$ = 0.09 I: *F*(2;66) = 3.56, *p* = 0.034, $\eta_{p}^{2}$ = 0.10
Subjective cognitive dysfunctions	9.45 (3.61)	9.76 (3.47)	8.35 (3.51)	8.35 (4.22)	8.90 (4.04)	9.20 (4.37)	G: *F*(2;66) = 0.71, *p* = 0.459, $\eta_{p}^{2}$ = 0.02 T: *F*(1;66) = 0.42, *p* = 0.520, $\eta_{p}^{2}$ = 0.01 I: *F*(2;66) = 0.10, *p* = 0.904, $\eta_{p}^{2}$ = 0.00
**Memory test**							
Hits	13.21 (2.35)	13.00 (2.41)	13.63 (2.09)	12.47 (1.65)	14.00 (1.12)	13.05 (1.23)	G: *F*(2;65) = 0.58, *p* = 0.563, $\eta_{p}^{2}$ = 0.02 T: *F*(1;65) = 6.86, *p* = 0.011, $\eta_{p}^{2}$ = 0.09 I: *F*(2;65) = 1.08, *p* = 0.346, $\eta_{p}^{2}$ = 0.03
False memories	1.00 (1.79)	1.38 (2.08)	0.73 (1.24)	1.32 (1.86)	0.45 (0.60)	1.85 (2.81)	G: *F*(2;65) = 0.60, *p* = 0.942, $\eta_{p}^{2}$ < 0.01 T: *F*(1;65) = 11.55, *p* = 0.001, $\eta_{p}^{2}$ = 0.15 I: *F*(2;65) = 1.85, *p* = 0.166, $\eta_{p}^{2}$ = 0.05
All high-confident responses	25.93 (5.79)	24.34 (6.94)	24.42 (5.91)	22.42 (5.62)	27.65 (3.38)	23.50 (6.21)	G: *F*(2;65) = 1.05, *p* = 0.356, $\eta_{p}^{2}$ = 0.03 T: *F*(1;65) = 11.34, *p* = 0.001, $\eta_{p}^{2}$ = 0.15 I: *F*(2;65) = 1.08, *p* = 0.345, $\eta_{p}^{2}$ = 0.03
**Psychopathology**							
LSHS-R	29.39 (8.37)	29.00 (8.51)	28.85 (9.45)	28.40 (9.99)	28.00 (10.43)	29.25 (11.77)	G: *F*(2;65) = 0.03, *p* = 0.971, $\eta_{p}^{2}$ = 0.00 T: *F*(1;65) = 0.09, *p* = 0.765, $\eta_{p}^{2}$ = 0.00 I: *F*(2;65) = 1.44, *p* = 0.243, $\eta_{p}^{2}$ = 0.04
Paranoia Checklist	40.03 (17.88)	37.55 (16.86)	38.00 (16.67)	32.85 (13.79)	31.35 (17.23)	30.60 (16.59)	G: *F*(2;66) = 1.37, *p* = 0.261, $\eta_{p}^{2}$ = 0.04 T: *F*(1;66) = 8.33, *p* = 0.005, $\eta_{p}^{2}$ = 0.11 I: *F*(2;66) = 1.62, *p* = 0.205, $\eta_{p}^{2}$ = 0.05
CES-D	55.07 (14.49)	50.66 (14.32)	56.75 (13.49)	52.40 (16.30)	47.75 (15.98)	47.15 (15.75)	G: *F*(2;66) = 1.42, *p* = 0.250, $\eta_{p}^{2}$ = 0.04 T: *F*(1;66) = 6.40, *p* = 0.014, $\eta_{p}^{2}$ = 0.09 I: *F*(2;66) = 1.00, *p* = 0.373, $\eta_{p}^{2}$ = 0.03


*CES-D, Center for Epidemiologic Studies-Depression Scale; LSHS-R, Launay-Slade Hallucination Scale-Revised; G, main effect of group, T, main effect of time, I, interaction effect of group and time.*

### Pre versus Follow-Up

**FIGURE 2:**
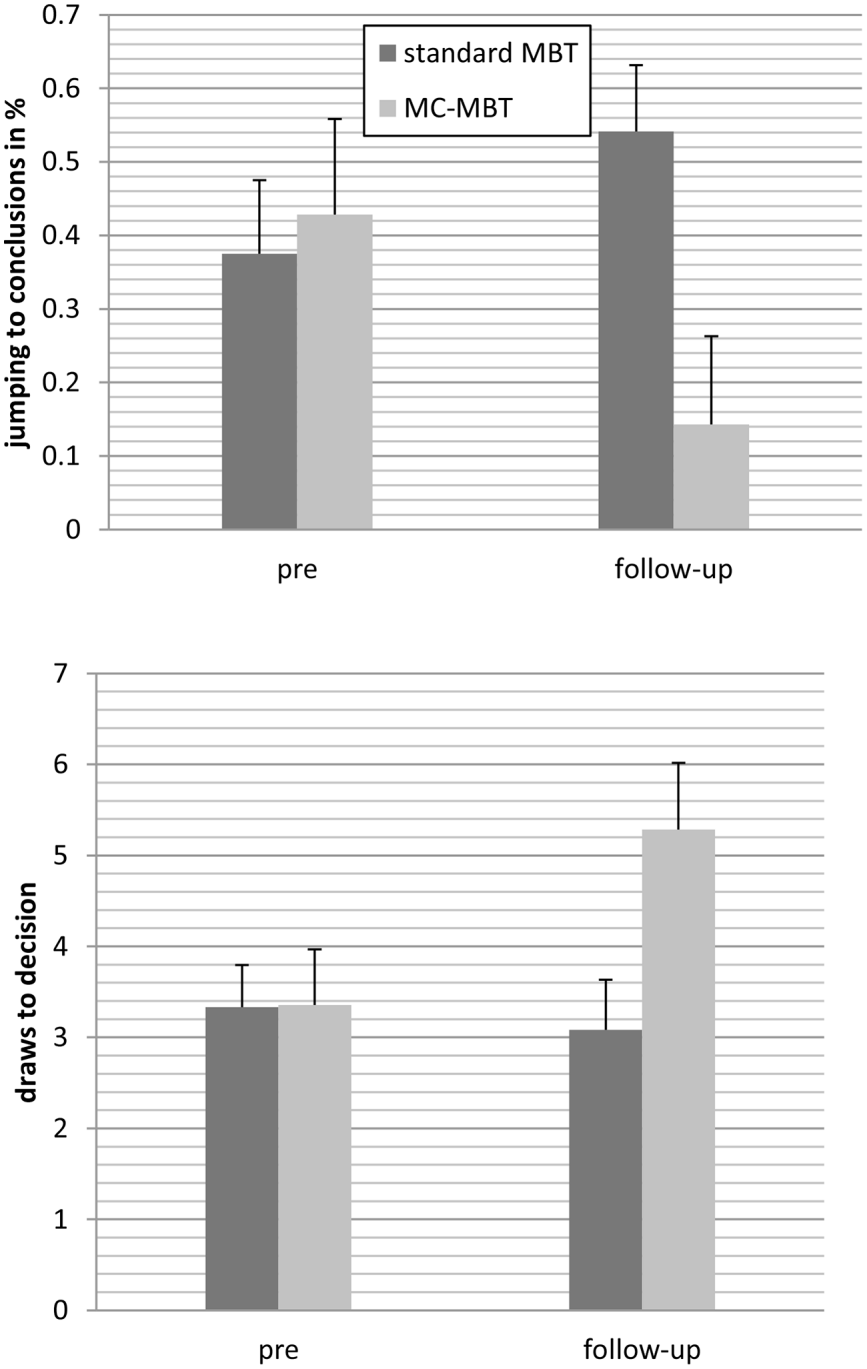
**Patients who underwent the metacognition-augmented cognitive remediation program (MC-MBT) showed less jumping to conclusions from baseline to follow-up (upper) and delayed decision-making (lower) relative to participants who received the standard version (MBT; immediately or delayed), respectively**.

At follow-up, 38 individuals underwent another assessment [metacognition-augmented mybraintraining: *n* = 14; standard mybraintraining (immediate or delayed): *n* = 24]. For draws to decision, the effect of time achieved statistical trend level, *F*(1;36) = 3.46, *p* = 0.071, $\eta_{p}^{2}$ = 0.09, while the group effect was insignificant, *F*(1;36) = 2.44, *p* = 0.127, $\eta_{p}^{2}$ = 0.06. This was qualified by a significant interaction, *F*(1;36) = 5.82, *p* = 0.021, $\eta_{p}^{2}$ = 0.14; **Figure 2** shows that participants in the metacognition-augmented condition showed delayed decision-making while participants in the standard condition showed a tendency toward more jumping to conclusions. Likewise, using generalized estimating equations to fit a repeated-measures logistic regression to jumping to conclusions data (decision after fish 1 or 2), a significant interaction occurred favoring the metacognition-augmented condition, Wald χ^2^(1) = 4.55, *p* = 0.033.

**FIGURE 3:**
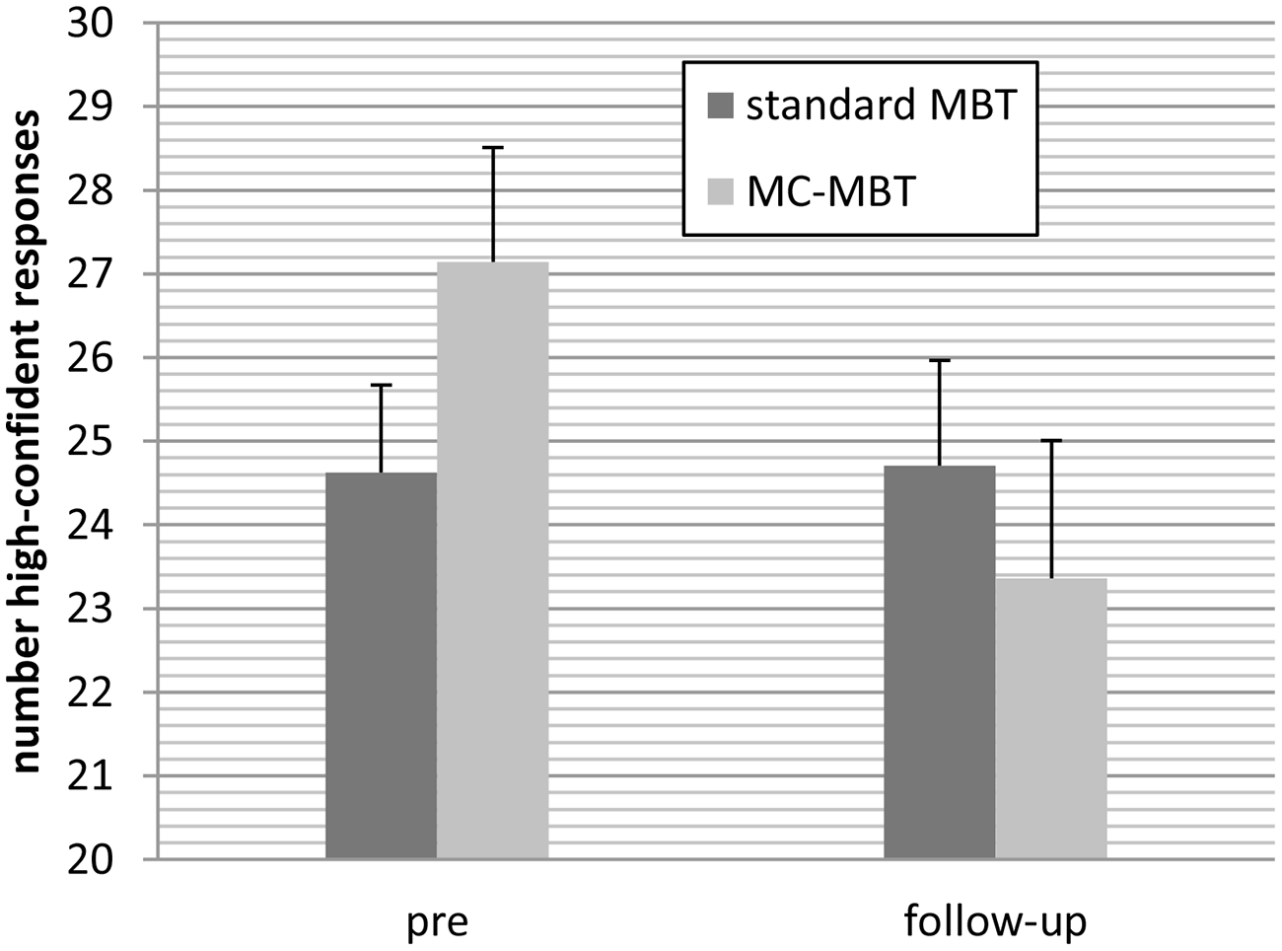
**Patients in the metacognition-augmented cognitive remediation condition (MC-MBT) attenuated confidence ratings from baseline to the follow-up period relative to the standard CR group (MBT)**.

For the number of high-confident responses on the memory test the effect of time, *F*(1;36) = 5.12, *p* = 0.030, $\eta_{p}^{2}$ = 0.125 but not group, *F*(1;36) = 0.11, *p* = 0.737, $\eta_{p}^{2}$ < 0.01 were significant, which was qualified by significant interaction at an almost large effect size, *F*(1;36) = 5.59, *p* = 0.024, $\eta_{p}^{2}$ = 0.13. As can be seen in **Figure 3** the number of high-confident responses remained stable in the standard CR group but declined in the metacognition-augmented group.

No significant interaction emerged for depression, *F*(1;36) = 0.14, *p* = 0.91, $\eta_{p}^{2}$ < 0.01, paranoia, *F*(1;36) = 0.64, *p* = 0.428, $\eta_{p}^{2}$ = 0.02, hits, *F*(1;36) = 0.78, *p* = 0.785, $\eta_{p}^{2}$ < 0.01, and false memories, *F*(1;36) = 1.23, *p* = 0.276, $\eta_{p}^{2}$ = 0.03.

### Retrospective Assessment (Post)

Feasibility and comprehensibility of the training were rated high by respondents and did not differ between the two CR conditions (**Table 3**). Patients were able to perform the tasks alone and rated the exercises as helpful, although only a minority reported symptom improvement.

**Table 3:** Retrospective subjective assessment (“fully applies” and “rather applies” were combined) at post.

Variable	Standard cognitive remediation	Metacognition-augmented cognitive remediation	Statistics
	(*n* = 20)	(*n* = 20)	
Program is suitable for self-administration.	95%	100%	1.13, *p* = 0.567
The instructions of the program were understandable.	85%	85%	1.90, *p* = 0.911
I considered the exercises as helpful.	65%	70%	0.70, *p* = 0.704
I was able to regularly perform the exercises in the past weeks.	60%	70%	1.18, *p* = 0.554
I had to force myself to perform the program regularly.	25%	45%	2.05, *p* = 0.359
The extent of the training was just right.	75%	60%	1.33, *p* = 0.515
Other persons have helped me with the program.	10%	0%	10.06, *p* < 0.001
I think the training is more appropriate in the framework of a psychotherapy.	35%	15%	4.51, *p* = 0.105
I could integrate the lessons learnt into my daily routine.	45%	20%	4.30, *p* = 0.116
Symptoms have decreased due to the program.	30%	20%	3.28, *p* = 0.149

### Correlations between Performance and Adherence with Symptomatology

We examined whether adherence and progress on the CR program impacted outcome variables. Progress in performance in CR memory exercises (slope change measure) was correlated at *r* = 0.61 (*p* = 0.026) with improvement in the memory test from pre to post for the standard mybraintraining group (no other variables turned significant). Gain in overall performance (all exercises combined) in the metacognition-augmented mybraintraining condition correlated with more draws to decision in the fish task, *r* = 0.54, *p* = 0.021 and less jumping to conclusions at trend level, *r* = -0.42, *p* = 0.079. Similarly, the number of exercises performed (objective measure) in the metacognition-augmented mybraintraining condition correlated with less jumping to conclusions significantly (*r* = -0.398, *p* = 0.040) and less draws to decision over time at trend level (*r* = 0.353, *p* = 0.071). Adherence in the standard condition (number of days the CR program was used) was associated with reduction of depression over time (*r* = 0.467, *p* = 0.028). Likewise, number of exercises performed (objective) was correlated with decline of depressive symptoms (*r* = 0.482, *p* = 0.023), again for the standard version only.

### Test–Retest Reliability of the Data and Plausibility Checks

Test–retest reliability was determined for pre–post scores only due to the low number of participants at follow-up. Consistency of the psychopathological scales was excellent (CES-D: pre–post: *r* = 0.817, *p* < 0.001; Paranoia Checklist: *r* = 0.891, *p* < 0.001, LSHS-R: *r* = 0.936, *p* < 0.001). The recognition test showed low reliability from pre to post (*r* = 0.255, *p* = 0.024). The correlation between subjective adherence (number of days exercises were performed: 0–7 days/week) and objective number of exercises performed (data extracted from log files) was good (*r* = 0.817, *p* < 0.001).

## Discussion

The study set out to examine the effectiveness of conventional as well as metacognition-augmented CR training. Most patients were on antipsychotic medication and in outpatient treatment. Treatment status did not change substantially across time. Special precautions were taken to verify diagnostic status. Speaking for the quality of the data, the test-retest reliability of the questionnaires was very high. Further, subjective and objective adherence were highly correlated.

We used a low-threshold online CR training termed mybraintraining targeting four cognitive domains which according to the developers (personal communication, unpublished data) are linked with metabolic changes in frontal lobe areas. Patients carried out the exercises on their home computer. The program was delivered unguided; no individual adaption was performed apart from automatic adjustments pertaining to difficulty. Our hypotheses were partly confirmed. Group comparisons indicate that conventional CR did not impact any outcome measure suggesting that *cold* cognitive functioning is quite resistant to cognitive training interventions, at least in a rather chronic and subacute psychosis population. At the same time, the CR version showed some interesting correlations with depression: the number of completed sessions was correlated with a reduction on the CES-D which could hint at (but is no proof for) the possibility that training improves well-being. This would be a potentially important finding as neither antipsychotic (Leucht et al., 2009) nor antidepressant medication (Kishi and Iwata, 2014) exert substantial effects on depression in psychosis. Likewise, psychotherapy with cognitive-behavioral therapy only yields a small-to-medium effect according to a meta-analysis (Wykes et al., 2008). However, an opposite causal relationship cannot be fully dismissed: Improvement of well-being may enhance fidelity to perform the tasks. Further, performance gains on the memory task were correlated with improvements on the objective memory test, speaking for the ecological validity of the task. Again, however, group differences were not significant.

At follow-up, the metacognition-enhanced CR training yielded the expected significant effects on the JTC bias (i.e., delayed decision-making) and reduced overconfidence. These findings are noteworthy since both biases are implicated in the formation of psychosis and JTC is rather resistant to change (Ross et al., 2011; So et al., 2012a,b). This delayed effect is interesting and may indicate that the newly acquired skills need some time to settle before they become manifest. At post, we found substantial correlations between fish task parameters with adherence and performance gain.

At first sight, the results are sobering in face of recent reviews indicating that CR tasks may yield at least small-to-medium effects on objective neurocognitive functioning (McGurk et al., 2007; Wykes et al., 2011). A number of factors may have prevented the hypothesized pattern of results from emerging. First, the training was self-paced, that is, individuals were encouraged to perform the tests daily but in fact many did not perform the tasks on a regular basis. In contrast, in many clinical trials on CR there are frequent appointments and homework is checked by therapists. A certain (cued) participation frequency may be necessary to show an effect. Our weekly email reminders may not have been sufficiently strong cues. Second, the group was not severely ill (mainly outpatient treatment) and self-help was performed predominantly at home as patients were not hospitalized. A chronic and more remitted sample is likely to show less benefit from training than an acute and hospitalized sample (e.g., because of regression to the mean). Thus, a first-episode and CR-naive treatment group may show better outcome. Third, the outcome measures did not cover the full range of domains targeted. In fact, we had only one objective memory test with rather low reliability. Perhaps the training exerted effects on functions not covered by our battery. Future studies should therefore administer a wider range of behavioral tests. Finally, while the initial sample was rather large and we had a good retention rate for the post phase, less than 50% participated in the follow-up.

## Conclusion

The metacognition-enhanced CR condition showed delayed changes on two prominent cognitive biases which are implicated in the pathogenesis of positive symptoms: jumping to conclusions and overconfidence. The program under investigation now incorporates these additional metacognitive features which are deemed important as prior studies suggest that JTC is quite resistant to change (see above) and is not only tied to positive symptoms but predicts functional outcome to some degree (Andreou et al., 2014). It seems that the training – like metacognitive training (MCT; Moritz et al., 2014a) – successfully “sows the seeds of doubt.” Further studies should investigate whether this leads to a reduction of symptoms in the long run.

## Conflict of Interest Statement

The authors declare that the research was conducted in the absence of any commercial or financial relationships that could be construed as a potential conflict of interest.
